# Thermal surface free energy and stress of iron

**DOI:** 10.1038/srep14860

**Published:** 2015-10-06

**Authors:** Stephan Schönecker, Xiaoqing Li, Börje Johansson, Se Kyun Kwon, Levente Vitos

**Affiliations:** 1 Applied Materials Physics, Department of Materials Science and Engineering, Royal Institute of Technology, Stockholm SE-10044, Sweden; 2 Department of Physics and Astronomy, Division of Materials Theory, Uppsala University, Box 516, SE-75120, Uppsala, Sweden; 3 Graduate Institute of Ferrous Technology, Pohang University of Science and Technology, Pohang 790-784, Korea; 4 Research Institute for Solid State Physics and Optics, Wigner Research Center for Physics, Budapest H-1525, P.O. Box 49, Hungary

## Abstract

Absolute values of surface energy and surface stress of solids are hardly accessible by experiment. Here, we investigate the temperature dependence of both parameters for the (001) and (110) surface facets of body-centered cubic Fe from first-principles modeling taking into account vibrational, electronic, and magnetic degrees of freedom. The monotonic decrease of the surface energies of both facets with increasing temperature is mostly due to lattice vibrations and magnetic disorder. The surface stresses exhibit nonmonotonic behaviors resulting in a strongly temperature dependent excess surface stress and surface stress anisotropy.

Introduced by Gibbs, the surface free energy (*γ*) and the surface stress (*σ*), are the two essential macroscopic parameters that characterize the thermodynamic properties of crystalline surfaces^1^,^2^. Today, there is growing appreciation that both parameters play an important role in understanding various surface phenomena, e.g., faceting, roughening, segregation, surface reconstruction, crystal growth, adsorption, bottom-up self-organization, and surface melting^1^,^2^,^3^,^4^,^5^,^6^. Surface stress also bears enormous potential for prospective molecular sensing and actuation devices exploiting its sensitivity to changes of chemical bonding and morphology in the surface region^7^,^8^.

Although Langmiur^9^ pointed out already a century ago that rising thermal agitation of atoms in solids would lead to a reduced work of creation of surface (the surface energy), its temperature dependence and that of the surface stress are still largely unknown in the absence of direct measurements. Today, we face the situation that the polar dependence of *γ* and *σ* is accessible experimentally—the anisotropy of the surface energy is encoded in the equilibrium shape of 3D crystals—, but the determination of their absolute values often lacks reliability and accuracy hampered by experimental difficulties^1^,^2^,^3^,^10^,^11^. Available absolute surface energies were in most cases either obtained at temperatures slightly below the melting point (*T*_m_) where plastic flow occurs or were derived from the surface tension in the liquid phase (*γ*_LV_)^12^,^13^,^14^. Absolute surface stress has proved elusive for direct measurements so far, merely few data for noble metals were derived from surface stress induced lattice strain in nanometer-sized particles^11^.

In view of the experimental difficulties, the determination of surface energy and surface stress from theory has become a convenient alternative route^15^,^16^,^17^,^18^, although modeling has been essentially limited to the ground state (*T* = 0 K) hitherto. Semi-empirical approaches^12^,^19^ estimated configurational and vibrational contributions to the surface excess entropy for an “average” high-index surface facet to access *γ*(*T*). These estimations rely, however, on dependable surface-tension data and carry errors of unknown magnitude at low-temperatures due to the uncertainty in the extrapolation procedure based on certain assumptions for the temperature dependence, e.g., a linear dependence resembling the behavior of the surface tension (Eötvös rule)^19^.

Various thermodynamic integration (TI) methods in combination with off-lattice Monte Carlo or molecular dynamics (MD) simulations have been employed to compute the vibrational contribution to the surface parameters, both potential-based^20^,^21^,^22^ and by means of *ab inito* simulations^23^. Unfortunately, the applicability of most TI variants is limited to solid systems that do not undergo an allotropic or magnetic phase transition in the temperature interval of interest.

The phase stability and mechanical properties of Fe are strongly influenced by magnetism and temperature^24^,^25^,^26^. Magnetic long range order and magnetic correlations in Fe are responsible for the stability of the body-centered cubic (bcc) (*α*) phase up to 1189 K significantly above the Curie temperature (*T*_C_ = 1043 K). The face-centered cubic (fcc) phase is stable in the temperature interval 1189 K–1662 K, but iron eventually melts from the reoccurring bcc (*δ*) phase at 1807 K. Surface magnetism in Fe was shown to have a pronounced impact on the magnitudes of surface energy and surface stress in the ground state^17^,^27^.

This paper brings forwards an density-functional theory (DFT) description of *γ*(*T*) and *σ*(*T*) for the two most stable surface facets of bcc Fe [(001) and (110)]^18^,^28^. Free energies [*F* = *F*(*T*, *V*)] were computed for a surface subsystem and for a bulk subsystem, from which the surface energies, *γ* = *A*^−1^(*F*^surf^ − *F*^bulk^), and the surface stresses, *σ* = (2*A*)^−1^∂(*F*^surf^ − *F*^bulk^)/∂*ε*|_*ε* = 0_, were obtained^2^. Here, *ε* specifies an isotropic and elastic in-plane deformation of the surface with unit area *A* and *σ* represents half the trace of the surface stress tensor.

## Theoretical modeling

We rely on the adiabatic approximation shown to be applicable to Fe^29^,^30^ and model the individual vibrational, electronic, and magnetic excitations to the free energy, *F* = *E*_0_ + *F*_vib_ + *F*_el_ + *F*_mag_. *E*_0_ is the total energy of the ferromagnetic ground state. The vibrational free energy contribution (*F*_vib_) and the bulk thermal expansion were derived within the quasiharmonic approximation (QHA)^31^ in the ferromagnetically ordered state using a gradient-corrected exchange-correlation parameterisation (PBE)^32^. The force-constant matrix was obtained within the framework of density-functional perturbation theory^31^,^33^ as implemented in the projector-augmented wave method (VASP)^34^ and employing Phonopy^35^ to compute the phonon density of states (DOS) and *F*_vib_. The electronic (*F*_el_) and magnetic (*F*_mag_ = *E*_mag_ − *TS*_mag_) contributions to the free energy were obtained with the exact muffin-tin orbitals (EMTO) method and the full charge-density technique^36^,^37^,^38^ using PBE in conjunction with the coherent-potential approximation (CPA)^39^,^40^. *F*_el_ takes into account electronic excitations due to smearing of the Fermi-Dirac distribution^41^,^42^. The magnetic disorder effect on the total energy (*E*_mag_) was approximated by the partially disordered-local moment (PDLM) model described within the framework of CPA^43^,^44^. The PDLM model connects the ordered ferromagnetic phase with the disordered paramagnetic (DLM) phase without taking into account short range order^45^. Accordingly, the gradual loss of magnetic long-range order, characterised by the staggered magnetisation (*m*, 1 ≥ *m *≥ 0), is captured by a gradual concentration change (*x*, 0 ≤ *x* ≤ 0.5) of a random binary alloy with anti-parallel moment orientation, 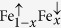, where *x* = 0 and *x* = 0.5 correspond to the ferromagnetic phase (*m* = 1) and the paramagnetic (DLM) phase (*m* = 0), respectively. To describe the loss of magnetisation as a function of temperature, *m* was mapped to *T* via an analytic representation (*m* = [1 − 0.35*τ*^3/2^ − 0.65*τ*^4^]^1/3^, *τ* = *T*/*T*_C_)^46^ of the experimental magnetisation curve of Fe^47^. Magnetic entropy (*S*_mag_) within the PDLM model was considered by the mean-field expression, *S*_mag_/*k*_B_ = 2*x* ln(*μ* + 1), where *k*_B_ and *μ* denote the Boltzmann constant and the local magnetic moment in units of Bohr magneton, respectively. We notice that the measured magnetic entropy of paramagnetic bcc iron is in agreement with this mean-field estimate^48^. Thermal lattice expansion was incorporated into the magnetic and electronic contributions via the lattice parameters obtained from the QHA for *F*_vib_.

For the surface energy *γ* full explicit phononic contributions and thermal expansion were considered. For the surface stress *σ*, we included the thermal expansion effect only. That is because previous investigations found that the dominating phononic contribution to the surface stress (of atomically ordered surfaces) arises from thermal expansion rather than from a temperature-induced change of the vibrational amplitude, even for open surface facets^21^. The computational details can be found in the Method’s section.

## Results and Discussion

**Figure 1:**
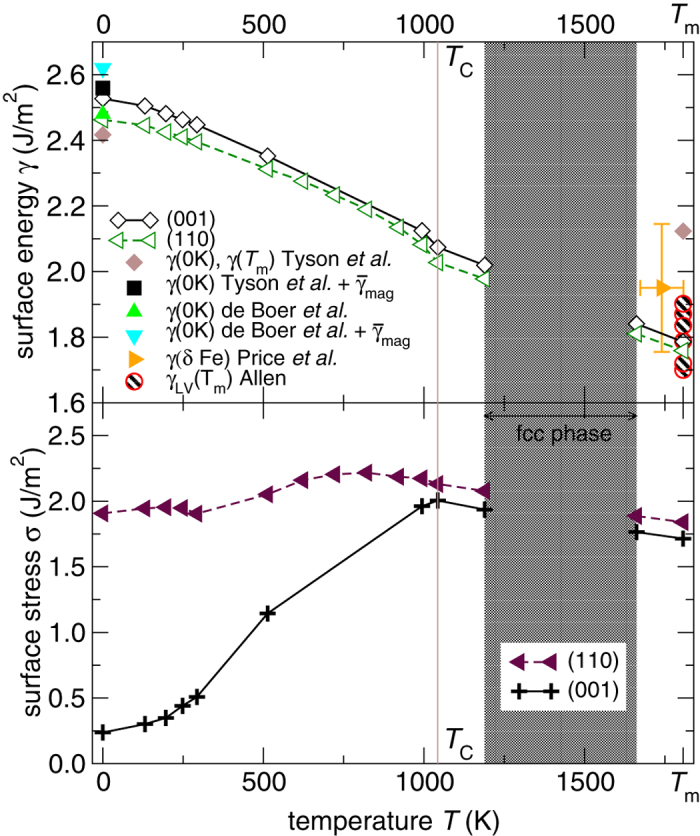
The calculated temperature effect on the surface energy and surface stress of the (110) and (001) facets of bcc Fe. Experimental surface energies of *δ* Fe were reported by Price *et al.*^14^, and the available surface tension measurements (*γ*_LV_) of liquid Fe were reviewed by Allen^13^. The surface energy at *T*_m_ was estimated by Tyson *et al.*^12^ and at 0 K by Tyson *et al.*^12^ and de Boer *et al.*^19^. The latter two 0 K estimates amended by the missing magnetic contribution 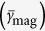 are also plotted and discussed in the text. Data from refs 12,14,19 should be understood as to represent an average surface facet. Lines guide the eye. We estimate the error of the computed surface energy (surface stress) at 0 K and *T*_m_ to <0.1 J/m^2^ (<0.2 J/m^2^) and 0.1–0.2 J/m^2^ (0.3–0.4 J/m^2^), respectively.

Figure 1 shows the computed temperature dependence of surface free energy and surface stress for the (001) and (110) surface facets of bcc Fe. Rising temperature from 0 K to *T*_m_ leads to a monotonically decreasing surface energy for both faces, i.e., by 28% and 29% for (110) and (001), respectively. The close-packed (110) surface facet maintains the lower surface energy in the entire stability range of the bcc phase, but the anisotropy *γ*_(110)_/*γ*_(001)_ slightly decreases with increasing temperature.

The surface stress of the (001) and (110) facets exhibits a nonmonotonic dependence on *T* (Fig. 1). While *σ*_(001)_ is significantly lower than *σ*_(110)_ in the fully-ordered magnetic state at 0 K, they approach each other with increasing temperature mainly due to a significant increase of the surface stress of the (001) facet. The surface stresses of the two facets are nearly of the same magnitude in the paramagnetic phase.

**Figure 2:**
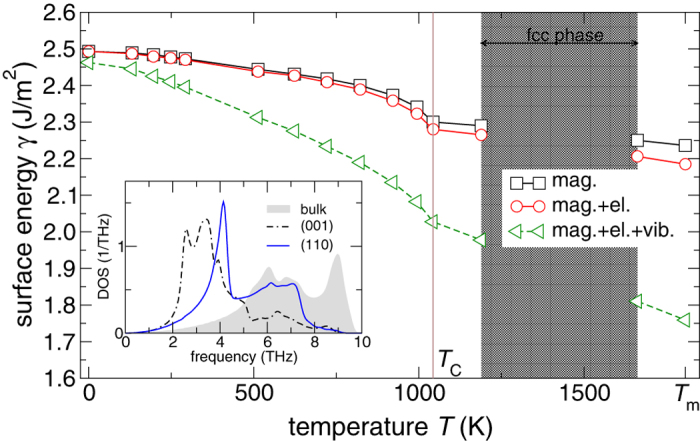
Decomposition of magnetic, electronic, and vibrational contributions to *γ*_(110)_(*T*) for the (110) surface facet of bcc Fe. The inset shows the phonon DOSs for the (110) and (001) surface facets in comparison to the bulk phonon DOS.

Figure 2 shows how the individual magnetic, electronic, and vibrational contributions add up to the surface free energy change as a function of temperature for the (110) surface facet. Accordingly, lattice vibrations reduce the surface energy most significantly followed by magnetic disorder. The magnetic disorder contribution to *γ* below *T*_C_ originates mainly from the continuous loss of magnetic long range order, while the magnetic entropy determines the magnetic contribution above *T*_C_. The overall small electronic disorder effect becomes significant only at temperatures higher than several hundreds of Kelvin. We note that the relative magnitudes of the individual contributions to *γ* are similar for the (001) surface facet. The absolute vibrational (magnetic) contribution to the surface free energy is, however, larger (smaller) for the open (001) facet than for the close-packed (110) facet. The different contributions due to phonons can be understood by comparing the phonon DOS for the two surface atomic layers with the bulk one as shown in the inset of Fig. 2 (DOSs computed for the equilibrium geometry). The reduced coordination number at both surface leads to an enhanced DOS at low phonon frequencies and a lower Debye temperature compared to the bulk counterpart. This softening of the vibrational modes is more pronounced for the (001) surface of Fe than for the (110) one^49^ resulting in a larger excess entropy at the more open surface facet.

In contrast to the monotonic trends seen for *γ* and the weakly temperature dependent *σ*_(110)_, *σ*_(001)_ increases significantly between 0 K and *T*_C_ (Fig. 1) resulting in a strongly temperature-dependent surface stress anisotropy (*σ*_(110)_/*σ*_(001)_). We analyzed this striking behavior by considering the magnetic contribution (*σ*_mag_) to the surface stress, where *σ*_mag_ is defined as the difference between the nonmagnetic value of the surface stress (no spin-polarization considered) and the surface stress from Fig. 1 (spin-polarization considered). The geometry of the lattice was kept fixed in this additional study. According to Punkkinen *et al.*’s^17^ analysis for magnetic metals, *σ*_mag_ was found to be proportional to the surface moment enhancement (Δ*μ*^2^), viz., 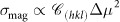, where 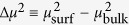. The surface magnetic moment (*μ*_surf_) of Fe is larger than the bulk one (*μ*_bulk_) leading to a compressive *σ*_mag_, i.e., *σ*_mag_ tends to expand the lattice and to reduce the total tensile surface stress^17^. Here, we introduced the facet-dependent positive proportionality constant 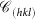 to reflect the influence of atomic coordination and surface electronic structure on *σ*_mag_. Δ*μ*^2^ for the open (001) facet was obtained by adding up the contributions from both the surface layer and the first subsurface layer, since *μ* differed significantly from *μ*_bulk_ in these two layers.

**Figure 3:**
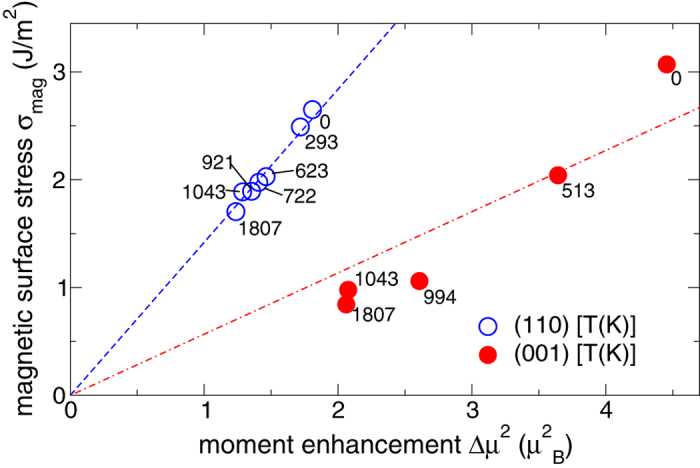
Correlation between moment enhancement and magnetic surface stress as a function of temperature (indicated by labels to the data points) on bcc (110) and (001) surface facets of Fe. The proportionality constants obtained from a fit to the data are 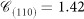 and 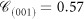.

The proportionality between *σ*_mag_ and Δ*μ*^2^ shown in Fig. 3 is excellent for the (110) surface facet and describes somewhat less satisfactorily the data of the (001) facet. *σ*_mag_ is nearly constant above *T*_C_ due to the small influence of thermal expansion on *σ*_mag_ and Δ*μ*^2^ in the DLM state. We find that a temperature increase from 0 K to *T*_m_ has a more significant effect on Δ*μ*^2^ for the (001) facet than for the (110) facet. This is plausible since the magnetic moments in the surface region of the densely packed (110) facet are smaller and converge more rapidly towards the bulk value compared to the situation at the open (001) facet^18^. We also observe that 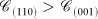 indicating that the enhanced surface magnetic moment for the (110) surface facet generates a larger surface stress than in the case of (001). Taking into account that the unit area per surface atom for (110) is a factor of 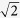 smaller than for (001), the magnetic surface stress per atom for (001) is still ≃43% smaller than that for (110). A possible explanation lies in the lower coordination number, and hence the smaller *d*-bandwidth, for an Fe atom situated at the (001) surface compared to when it is located at the (110) surface. Since 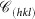 is inversely proportional to the height of the single-particle DOS^17^,^50^, 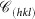 should roughly scale with the width of the *d*-band. Thus, the difference in the surface atom coordination number confirms our finding 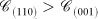.

Available experimental data for *γ* of *δ* Fe allows for a comparison with the present *ab initio* surface energies. Price reported *γ* = 1.95 J/m^2^ ± 10% in the temperature range 1673 K – *T*_m_^14^. Our prediction for *γ* slightly below *T*_m_ lies within the uncertainty of the experiment, see Fig. 1. The jump of *γ* across the liquid-solid phase transition may further be used to estimate *γ*(*T*_m_) from *γ*_LV_ adding to it the latent heat of fusion per surface area, viz., *γ*(*T*_m_) ~ Δ*H*_f_/*A* + *γ*_LV_^51^. Using available values for Fe, Δ*H*_f_ = 13.81 kJ/mol^52^ and *γ*_LV_ = 1.78 ± 0.08 J/m^2^ 13, we arrive at 2.04 ± 0.08 J/m^2^ for the (001) facet and at 2.15 ± 0.08 J/m^2^ for the (110) facet. These values are somewhat larger than our predicted surface energies at *T*_m_, *γ*_(110)_ = 1.76 J/m^2^ and *γ*_(001)_ = 1.79 J/m^2^. A possible reason for the underestimation of *γ* near *T*_m_ could be that the vibrational contribution to *γ* for paramagnetic Fe is somewhat overestimated by the present theory. An estimation of the influence of the magnetic state of Fe on the vibrational contributions to the surface energy can be found in the Supplementary Information online. For the (110) surface facet, we found that the surface vibrational excess is nearly identical in the two magnetic phases. This indicates that the here reported vibrational contribution to *γ*_(110)_ is not expected to be significantly altered by the magnetic state of Fe. For the (001) surface facet, we estimated that the surface vibrational excess to *γ* in the paramagnetic state is ≃40% smaller than in the ferromagnetic state. This corresponds to an underestimation of *γ*_(001)_ by 0.1 J/m^2^ at *T*_m_. The updated estimate for *γ*_(001)_(*T*_m_), accounting for the reduced vibration excess in the paramagnetic state of Fe, is 1.89 J/m^2^ in closer agreement with the experimental data from Price^14^ and the above value derived from *γ*_LV_.

Semi-empirical estimates of *γ*(0 K) for an average surface facet, derived from experimental surface-tension data and estimated configurational and vibrational contributions, are available for comparison: *γ*(0 K) = 2.42 J/m^2^ from ref. 12 based on a nonlinear extrapolation scheme, and *γ*(0 K) = 2.48 J/m^2^ from ref. 19 assuming that *γ* is a linear function of *T*. Magnetic disorder was not considered in either approach, although the decomposition of *γ*(*T*) (Fig. 2) suggests that the magnetic part should be taken into account for a more accurate low temperature estimate. We quantified the magnetic part for an average surface facet on the basis of the presently calculated magnetic contribution to the surface energy. To this end, the magnetic contributions to *γ*_(001)_ and *γ*_(110)_ in the range 0 K to *T*_m_ were weighted by their fractional areal contributions to the equilibrium shape of a 3D crystallite according to the Wulff construction^10^ (other crystal faces possessing larger surface energies were not considered, as they contribute only by small fractions to the equilibrium shape^18^). Using the present 0 K values, *γ*_(110)_ = 2.46 J/m^2^ and *γ*_(001)_ = 2.53 J/m^2^, the fractions of {001} facets and of {110} facets to the equilibrium crystal shape are 0.23 and 0.77, respectively. This gives an average magnetic contribution 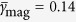 J/m^2^, which when added to the aforementioned estimated figures results in 2.56 J/m^2^ (with the value from ref. 12) and 2.62 J/m^2^ (ref. 19) respectively. The former estimate is in very good agreement with the present values.

A possible measure to indicate surface reconstruction is based on a bulk continuum elastic model, viz., 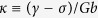  0.1–0.2, where the upper limit corresponds to more corrugated surface facets^1^,^53^. Accordingly, the larger is the excess surface stress (*γ* − *σ*), or the smaller is the shear modulus (*G*) [the length of the Burger’s vector (*b*) is nearly constant], the higher is the tendency to reconstruct. The predicted trends for *γ* and *σ* (Fig. 1) indicate that the excess surface stress and hence the propensity to reconstruct would decrease with rising temperature for both surface facets. However, the observed strong softening of the shear elastic constants with increasing temperature^24^,^25^ works against the excess surface stress. Expressing *G* through the Hill average^54^ of the experimental single-crystalline elastic constants from refs 24,25 and evaluating *κ* for *α* Fe in the ferromagnetic and paramagnetic phases, we found that *κ* decreases as *T* increases. The largest *κ* values (at 0 K) are 0.09 and 0.02 for the (001) facet and the (110) facet, respectively, indicating stable surfaces. This prediction is in line with observations, namely, no surface reconstruction was observed for the (110) and (001) facets of Fe in the temperature range 300–500 K^55^.

To conclude, temperature has a pronounced effect on the surface energy and the surface stress of bcc Fe. The surface energies of the (001) and (110) facets decrease nonlinearly by approximately 30% from 0 K up to the melting point with slight reduction of their anisotropy. This reduction of the work of separation is primarily due to lattice vibrations and magnetic disorder. There is good agreement between the present surface energies and the 0 K values from Tyson *et al.*’s and de Boer *et al.*’s databases^12^,^19^ especially if the latter values are corrected for the missing magnetic term. The surface stresses are nonmonotonic functions of temperature exhibiting a strongly temperature dependent surface stress anisotropy. Surface stress originating from the thermal magnetic disorder at the (001) surface is more strongly affected by temperature than the one at (110) and is mainly responsible for the pronounced increase of the stress at (001) below the Curie temperature. We predict that the propensity for surface reconstruction at (001) and (110) lowers as temperature increases.

Today, the theoretical description of metal surfaces at elevated temperature is a very challenging task. In order to make such a study feasible in the case of complex systems, one has to adopt several simplifications and approximations. Despite of that, the present theoretical results turn out to follow closely the previous semi-empirical estimates, demonstrating the predictive power of our *ab initio* tools in the case of high-temperature surface properties. These finding offers a solid platform for extending the existing theoretical methodology to study the surface properties of technological alloys.

## Methods

Convergence of all numerical parameters was carefully checked. Bulk and surface reference systems were of identical size to ensure numerical error cancellation. *F*_vib_ for the surface subsystem was obtained from 32–34 Å thick slabs separated by 10–14 Å vacuum. The relaxation at 0 K included all interlayer distances perpendicular to the surfaces until the residual forces on each layer were smaller than 0.05 meV/Å. To compute the force constant matrix (VASP), we used a plane wave cut-off of 500 eV and a *k*-point mesh equivalent to a 24 × 24 × 2(1) Monkhorst-Pack mesh of the primitive bulk (surface) reference system. The phonon properties were sampled on a 3 × 3 × 1 mesh. All derived vibrational quantities that involve an integration of the phonon density of states (i.e., free energies) are converged to within ±2% against a denser 4 × 4 × 1 sampling mesh. We checked the temperature-dependent surface inter-layer relaxation of Fe within the QHA according to ref. 56. We found that relaxation at the (110) facet, determined at 1043 K and at 1805 K, change *γ* and *σ* by <2% and by <4%, respectively. Since this relaxation effect represents only a small correction to both *γ* and *σ* at significantly higher computational cost, finite temperature surface inter-layer relaxation was not included in the present *F*_vib_, i.e., the relaxed *T* = 0 K interatomic distances were rigidly rescaled according to the bulk thermal expansion.

The magnetic and electronic disorder effects were modelled by slabs with a converged thicknesses of 13 atomic layers and 9 atomic layers for the (001) surface and the (110) surface, respectively, separated by ~10 Å vacuum. Surface energies and surface stresses calculated with EMTO were sampled on a 15 × 15 × 2(1) *k*-point mesh in the case of the bulk (surface) reference system.

## Additional Information

**How to cite this article**: Schönecker, S. *et al.* Thermal surface free energy and stress of iron. *Sci. Rep.* **5**, 14860; doi: 10.1038/srep14860 (2015).

## Supplementary Material

Supplementary Information
