# Supplementary material for: Thermal surface free energy and stress of iron
Source: Sci Rep. 2015 Oct 6;5:14860. doi: 10.1038/srep14860 (PMC4594358; doi:10.1038/srep14860)
Supplement: Supplementary Information [file srep14860-s1.pdf]

Supplementary information: Thermal surface free energy and stress of iron

Stephan Schönecker[1,*], Xiaoqing Li[1], Börje Johansson[1,2], Se Kyun Kwon[3], and Levente Vitos[1,2,4]

[1]Applied Materials Physics, Department of Materials Science and Engineering, Royal Institute of Technology, Stockholm SE-10044, Sweden
[2]Department of Physics and Astronomy, Division of Materials Theory, Uppsala University, Box 516, SE-75120, Uppsala, Sweden
[3]Graduate Institute of Ferrous Technology, Pohang University of Science and Technology, Pohang 790-784, Korea
[4]Research Institute for Solid State Physics and Optics, Wigner Research Center for Physics, Budapest H-1525, P.O. Box 49, Hungary
[*]stesch@kth.se

Influence of magnetic state on the vibrational contribution to the surface energy

We wish to estimate the influence of the ferromagnetic (fm) versus paramagnetic (pm) state of Fe on the vibrational contributions to the surface energy (γ) at high temperatures. To this end, we assume that a characteristic Debye temperature (θ_{D}) can be defined so that F_{vib} equals the free energy within the Debye model [$F_{\mathrm{D}}(\theta_{\mathrm{D}})$] at a given T. The existence of a surface perturbs the free energy of the bulk, i.e., $\Delta F_{\mathrm{D}} \equiv F_{\mathrm{D}}^{\mathrm{surf}}(\theta_{\mathrm{D}}^{\mathrm{surf}}) - F_{\mathrm{D}}^{\mathrm{bulk}}(\theta_{\mathrm{D}}^{\mathrm{bulk}}) \equiv A\gamma_{\mathrm{D}}$, where γ_{D} denotes the (Debye) vibrational contribution to the surface energy. In the limit $T > \theta_{\mathrm{D}}$, ΔF_{D} is determined by the entropy term, i.e., $\Delta F_{\mathrm{D}} \simeq -T\Delta S_{\mathrm{D}}$, where $\Delta S_{\mathrm{D}} \equiv S_{\mathrm{D}}^{\mathrm{surf}}(\theta_{\mathrm{D}}^{\mathrm{surf}}) - S_{\mathrm{D}}^{\mathrm{bulk}}(\theta_{\mathrm{D}}^{\mathrm{bulk}})$ and S_{D} denotes the Debye entropy.[1] It is readily shown that ΔS_{D} approaches a constants value,

$$\frac{\Delta S_{\mathrm{D}}}{k_{\mathrm{B}}} \simeq 3\ln\left(\frac{\theta_{\mathrm{D}}^{\mathrm{surf}}}{\theta_{\mathrm{D}}^{\mathrm{bulk}}}\right) \quad \text{for } T > \theta_{\mathrm{D}}^{\mathrm{bulk}} (> \theta_{\mathrm{D}}^{\mathrm{surf}}), \tag{1}$$

so that ΔF_{D} is essentially linear in T. The relative vibrational contribution to the surface energy (γ_{vib}) for the pm and fm states of Fe are then approximately given by (high temperature limit)

$$\frac{\gamma_{\mathrm{vib}}^{\mathrm{pm}}}{\gamma_{\mathrm{vib}}^{\mathrm{fm}}} \simeq \frac{\gamma_{\mathrm{D}}^{\mathrm{pm}}}{\gamma_{\mathrm{D}}^{\mathrm{fm}}} \simeq \beta \frac{\ln\alpha^{\mathrm{pm}}}{\ln\alpha^{\mathrm{fm}}}, \quad \alpha \equiv 1 + \frac{\theta_{\mathrm{D}}^{\mathrm{e}}}{\theta_{\mathrm{D}}^{\mathrm{bulk}}},\ \beta^{-1} = \frac{A^{\mathrm{pm}}}{A^{\mathrm{fm}}} \tag{2}$$

where $\theta_{\mathrm{D}}^{\mathrm{e}} \equiv \theta_{\mathrm{D}}^{\mathrm{surf}} - \theta_{\mathrm{D}}^{\mathrm{bulk}}$ represents a (negative) surface excess. For the fm state, α could be determined from a Debye fit to the low-frequency part of the phonon DOSs shown in Fig. 2 in the main document. Since the same data sets are, however, not available for the pm state, we consider instead the long-wavelength limit (sound waves), defined by elastic properties, to estimate the ratio of the Debye temperatures in Eq. (2).

The perturbation of elastic modes due to the existence of a surface is expressed by excess surface elastic constants (C^{e}),[2] which, assuming the same isotropic distortion as for the surface stress (main document), are defined by

$$C^{\mathrm{e}} = (2A)^{-1}\partial^2(E^{\mathrm{surf}} - E^{\mathrm{bulk}})/\partial\varepsilon^2|_{\varepsilon=0}. \tag{3}$$

Here, $E = E_0 + E_{\mathrm{mag}}$ accounts for magnetic disorder. The softening of the surface vibrational modes relative to the bulk is expressed by a negative excess, i.e., $C^{\mathrm{e}} < 0$. The surface excess of the elastic modes is confined to the depth of the surface zone (nd), here denoted by n and expressed in terms of the equilibrium interlayer distance (d). Splitting up the surface subsystem (first term on the right hand side of Eq. (3); which computationally corresponds to a slab) into the surface zone and a bulk-like zone, the total energy contribution originating from the bulk-like zone formally cancels the one from the bulk subsystem taken for the same number of layers (second term on the right hand side of Eq. (3)). The remaining part is the total energy excess of the surface zone with respect to the bulk of identical thickness (nd). We may then denote by nC^{bulk} the bulk part of C^{e} for the depth of the surface zone, where C^{bulk} is the bulk contribution per atomic layer. For transition metals, we found that $n \simeq 2-3$

is reasonable in agreement with previous assessments.[2–4] In the following, we use $n = 2$. Analogous to the bulk case,[5] where $\theta_D \propto B^{1/2}$, B being the bulk modulus, for the surface excess we have $\theta_D^e \propto -|C^e|^{1/2}$ and $\theta_D^{bulk} \propto (nC^{bulk})^{1/2}$. The final result for α reads $\alpha \simeq 1 - (|C^e|/2C^{bulk})^{1/2}$, which is used in conjunction with Eq. (2) to estimate γ_{vib}^{pm} from the computed γ_{vib}^{fm}.

We obtained for $(\gamma_{vib}^{pm}/\gamma_{vib}^{fm})_{(001)} \simeq 0.6$ and for $(\gamma_{vib}^{pm}/\gamma_{vib}^{fm})_{(110)} \simeq 1.0$. The former result indicates that the surface excess to the surface energy due to vibrations on the (001) surface facet is smaller in the pm state than in the fm state. The latter value for the (110) surface facet signals that the vibrational contribution to the surface excess is rather independent on the magnetic state. Finally, accounting for fm order below T_C and pm order above $\gamma(T_m)$ may be obtained by

$$\gamma(T_m) \approx \gamma^{fm}(T_C) + \left(\gamma^{fm}(T_m) - \gamma^{fm}(T_C)\right) \frac{\gamma_{vib}^{pm}}{\gamma_{vib}^{fm}}. \tag{4}$$

We notice that within the PDLM model (main document) we may obtain $\gamma(T)$ by an integration of the weighted fm and pm contributions, $2x(T)\frac{\partial \gamma^{pm}}{\partial T} + (1 - 2x(T))\frac{\partial \gamma^{fm}}{\partial T}$, $x(T)$ being the PDLM concentration mapped to T. Since $x(T)$ in the fm phase is significantly larger than 0 only in the vicinity of T_C, the so-weighted value for $\gamma(T)$ is only slightly smaller than the value directly obtained from Eq. (4).

References

1. Desjonquères, M. C. & Spanjaard, D. *Concepts in Surface Physics* (Springer-Verlag, Berlin, 1998), 2nd edn.
2. Müller, P. & Saúl, A. Elastic effects on surfaces physics. *Surf. Sci. Rep.* **54**, 157 (2004).
3. Filippetti, A. & Fiorentini, V. Theory and applications of the stress density. *Phys. Rev. B* **61**, 8433–8442 (2000). URL http://link.aps.org/doi/10.1103/PhysRevB.61.8433.
4. Feibelman, P. J. Calculation of surface stress in a linear combination of atomic orbitals representation. *Phys. Rev. B* **50**, 1908–1911 (1994). URL http://link.aps.org/doi/10.1103/PhysRevB.50.1908.
5. Moruzzi, V. L., Janak, J. F. & Schwarz, K. Calculated thermal properties of metals. *Phys. Rev. B* **37**, 790–799 (1988). URL http://link.aps.org/doi/10.1103/PhysRevB.37.790.
